# Qualitative and quantitative analysis data of the major constituents of *Ilex paraguariensis* leaves by UPLC-PDA and QTOF-MS

**DOI:** 10.1016/j.dib.2016.05.022

**Published:** 2016-05-20

**Authors:** Carlos Henrique Blum-Silva, Ana Beatriz Gobbo Luz, Marcus Vinicius P.S. Nascimento, Bruno Matheus de Campos Facchin, Bruna Baratto, Tânia Silvia Fröde, Louis Pergaud Sandjo, Eduardo Monguilhott Dalmarco, Flávio Henrique Reginatto

**Affiliations:** a Departamento de Ciências Farmacêuticas, Centro de Ciências da Saúde, Universidade Federal de Santa Catarina, 88040-900 Florianópolis, Brasil; b Departamento de Análises Clínicas, Centro de Ciências da Saúde, Universidade Federal de Santa Catarina, 88040-900 Florianópolis, Brasil; c Department of Molecular Biotechnology and Health Sciences, Molecular Biotechnology Center, University of Torino, 10124 Torino, Italy

**Keywords:** *Ilex paraguariensis*, Mate, Chemical composition, LC-PDA, LC-MS

## Abstract

*Ilex paraguariensis* A. St. Hil. is a native plant of South America widely consumed as beverages for its ethno pharmacological properties, such as antioxidant, anti-inflammatory, hypocholesterolemic as well as its benefits on the cardiovascular system. Since these properties are related to its chemical composition, the identification and quantification of the major compounds of *I. paraguariensis* extracts still remains relevant. The data described in this article supports previous results on the anti-inflammatory effect of *I. paraguariensis* A. St. Hil (Mate), “The anti-inflammatory effect of *I. paraguariensis* A. St. Hil (Mate) in a murine model of pleurisy” [1]. The present data article reports on nine major compounds identified in *I. paraguariensis* extracts and its related fractions by using UPLC-PDA and UPLC-QTOF. Identification of the constituents was based on their retention times, UV absorption spectra and mass spectra data, as well as by comparison with authentic samples. The validated parameters show that the quantification by UPLC-PDA methodology developed is sensitive, precise and accurate.

**Specifications Table**

**Table:** 

Subject area	*Pharmaceutical sciences*
More specific subject area	*Natural product chemistry; Pharmacognosy*
Type of data	*Figure and tables*
How data was acquired	*Ultra performance liquid chromatography with photo-diode array and mass spectrometry detection using an Acquity-UPLC™ (Waters, MA, USA) coupled to a high-resolution mass spectrometer (Xevo G2-S QTof model), equipped with an electrospray ionization source and controlled by MassLynx v.4.1 software.*
Data format	*Analyzed*
Experimental factors	*Hydroethanolic extract of Ilex paraguariensis leaves was prepared by turboextraction. The extract was dried (CE) and a portion was poured onto water and partitioned with n-BuOH, yielding the n-BuOH fraction (BF) and aqueous residual fraction (ARF). For the LC analysis, the samples were dissolved in the mobile phase and filtered through a 0.22 µm membrane before the injection in UPLC.*
Experimental features	*The extract and the fractions of Ilex paraguariensis leaves were analyzed qualitatively and quantitatively by UPLC-PDA and UPLC-MS.*
Data source location	*Department of Pharmaceutical Science, Centre of Health Sciences, Federal University of Santa Catarina, 88040-900, Florianopolis, Brazil.*
Data accessibility	*Data is with this article*

**Value of the data**

• Chromatographic and mass spectrometric data can be used for comparison with other studies performed on *I. paraguariensis* extracts.
• The extract contents and the metabolites identification data will provide a valuable reference for studies comprising the chemical and pharmacological effects of *I. paraguariensis*.
• Allows the characterization of new targets and new potential functions for this medicinal plant.
• Interaction network generated.

## 1 Data

**Fig. 1:**
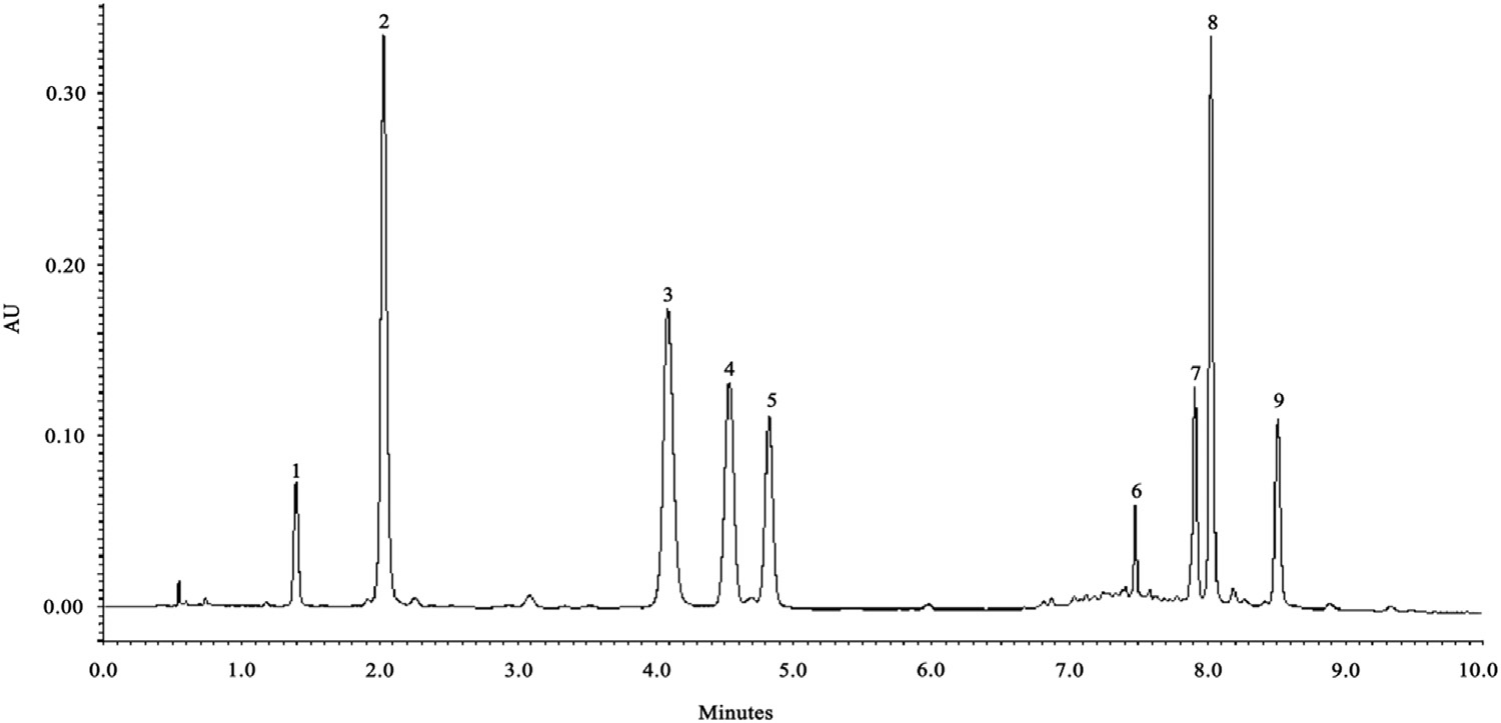
UPLC-PDA chromatogram at 280 nm. Peak identification: 1, theobromine; 2, 3-O-caffeoylquinic acid; 3, 5-O-caffeoylquinic acid; 4, caffeine; 5, 4-O-caffeoylquinic acid; 6, rutin; 7, 3,4-dicaffeoylquinic acid; 8, 3,5-dicaffeoylquinic acid; 9, 4,5-dicaffeoylquinic acid.

The following dataset includes one figure and two tables that support the identification as well as validation assays to quantify the major compounds of *Ilex paraguariensis* samples extracts. Fig. 1 shows the UPLC-PDA chromatographic profile of *I. paraguariensis* crude extract. The chromatographic and spectroscopic (UPLC-PDA and UPLC-MS) detection parameters data for each identified compound are presented in Table 1. Further, Table 2 data shows the validated parameters of the methodology developed. The different contents data of compounds of *I. paraguariensis* crude extract and its related fractions are showed in Table 1 in Ref. [1].

## 2 Experimental design, materials and methods

### 2.1 Plant material

Plant material data described here has been carried out in accordance with Ref. [1]. After harvesting, *I. paraguariensis* A. St. Hil. leaves (RSPF 11074) were processed and stored until the extraction and the preparation of its related fractions.

### 2.2 Extract preparation

The hydroethanolic *I. paraguariensis* crude extract were prepared by turboextraction in an Ultra-Turrax^®^ apparatus. After filtration, the solvent was removed under reduced pressure, and the obtained crude extract (CE) was partitioned between *n*-BuOH and water affording fractions BF and ARF, respectively. For details of the samples preparation see Ref. [1].

### 2.3 Chromatographic separation

All the chromatographic conditions are related to Ref. [1]. Chromatographic separation was achieved with an Acquity-UPLC™ (Waters^®^, MA, USA) system equipped with a quaternary pump, degasser, autosampler, photodiode array detector (PDA) and a Waters BEH C18 column, 1.7 μm, 50×2.1 mm at 40 °C as the stationary phase. The method used a gradient at constant flow rate (0.3 mL min^−1^) combining solvent A (formic acid/water, pH 2.5) and solvent B (acetonitrile), programmed as follows: 0-5 min, linear change from A–B (97:3 v/v) to A–B (90:10 v/v); 5–6 min, isocratic A–B (90:10 v/v); 6-9 min, linear change to A–B (80:20 v/v) and 9–10 min, linear change to A–B (10:90 v/v). The peaks were characterized by comparing the retention time, UV spectra and by co-injection of the sample with the reference standards. Quantification was performed by external calibration curve, using their corresponding standards. Caffeine (Sigma-Aldrich^®^) was quantified at 280 nm, while the phenolic compounds chlorogenic acid (Fluka^®^) and rutin (Sigma-Aldrich^®^) were quantified at 320 nm. All the analyses were performed in triplicate, and the peak area measured. Quantification was achieved using regression curves in the following ranges: 0.05–50 μg mL^−1^ for the caffeine; 0.05–100 μg mL^−1^ for chlorogenic acid and 0.25–50 μg mL^−1^ for rutin. The regression equations were “*y*=39320*x*+2074.9” for caffeine, “*y*=46696*x*−1896” for chlorogenic acid, and “*y*=15618*x*+178.92” for rutin. The extract was analyzed at a concentration of 500 μg mL^−1^ and the injection volume was 5 μL.

### 2.4 UPLC-MS analysis

In addition to UPLC-PDA analysis, the identification was carried out by liquid chromatography (UPLC, Waters^®^ Acquity mode) coupled to a high-resolution mass spectrometer (Xevo G2-S QTof model), equipped with an electrospray ionization probe. MassLynx v.4.1 software was used for data acquisition and processing. The mass spectrometer parameters were set as detailed in Ref. [1].

### 2.5 UPLC-PDA validation procedure

The UPLC-PDA quantification method was validated according to the ICH guidelines (2005) [6] for specificity, linearity, accuracy, precision (repeatability and intermediate precision), limit of quantification (LOQ) and limit of optical detection (LOD). The good linearity ranges were achieved by the analysis of linear correlation coefficient (caffeine, *r*^2^=1; chlorogenic acid, *r*^2^=0.9999; rutin, *r*^2^=0.9999) of the regression curves. The precision was determined by repeatability (intra-day assay) and intermediate precision (inter-day assay). The intra-day assay was performed in triplicate analysis of three different concentrations of the standard solutions, and expressed as relative standard deviation (RSD). The inter-day assay was determined by the analysis of a medium concentration in the curve, three times a day, on three different days. The limit of quantification (LOQ) and limit of detection (LOD) were defined by relative standard deviation (RSD<5%) and by a signal:noise ratio of 3:1, respectively. Accuracy was determined by spiking samples with the standard solutions of caffeine, chlorogenic acid or rutin (1:1 v/v) and the average recovery values were calculated.

## Figures and Tables

**Table 1:** Chromatographic and spectroscopic profile of phenolic compounds and methylxanthines of the extract and fractions from *Ilex paraguariensis* leaves.

**Peak**	**Identification**	**RT (min)**	***λ**_**max**_***(nm)**	**[M-H]**^**−**^**(m/z)**	**Error (mDa)**	a**Reference**
**1**	Theobromine	1.4	272	n.d.	–	Standard
**2**	3-O-caffeoylquinic acid	2.05	325–296	353.0903	−3,1	(An et al. [2]; Dartora et al. [3])
**3**	5-O-caffeoylquinic acid	4.1	325–296	353.0903	−1.3	Standard (Granica et al. [4]; Dartora et al. [3])
**4**	Caffeine	4.55	273	n.d.	–	Standard
**5**	4-O-caffeoylquinic acid	4.85	325–296	353.0903	0.3	Standard (Granica et al. [4]; Dartora et al. [3])
**6**	Rutin	7.5	353–255	609.1376	−5,4	Standard (An et al. [2]; Dartora et al. [3])
**7**	3,4-dicaffeoylquinic acid	7.91	325–296	515.1198	−2.0	(Li et al. [5]; Dartora et al. [3])
**8**	3,5-dicaffeoylquinic acid	8.05	325–296	515.1152	−6.9	(Li et al. [5]; Dartora et al. [3])
**9**	4,5-dicaffeoylquinic acid	8.52	325–296	515.1105	−10.2	(Li et al. [5]; Dartora et al. [3])

n.d. – not detected.

a Identification based on mass spectra, chromatographic profiles and previous reports.

**Table 2:** Validated parameters for the UPLC-PDA quantification.

**Compound**	**Precision**^a^				**Accuracy**^b^**(Recovery)**		**LOQ**^c^**(µg mL**^**−1**^**)**	**LOD**^c^**(µg mL**^**−1**^**)**
	**Repeatability**		**Intermediate precision**					
	**Mean (µg mL**^**−1**^**)**	**R.S.D. (%)**	**Mean (µg mL**^**−1**^**)**	**R.S.D. (%)**	**Mean (%)**	**R.S.D. (%)**		
**Caffeine**	0.05	3.17	10.00	1.01	100.9	1.07	0.05	0.015
	10.00	0.26						
	50.00	0.34						
**Chlorogenic acid**	0.05	4.77	50.00	1.02	99.5	1.17	0.05	0.01
	50.00	3.49						
	100.00	0.18						
**Rutin**	0.25	4.07	50.00	1.68	97.6	1.53	0.25	0.01
	10.00	3.37						
	50.00	0.82						

a Limit:R.S.D.<5%.

b Recovery was determined by injection of spiked samples, in triplicate, with standard solution.

c LOQ=limit of quantification; LOD=limit of detection.
