# Supplementary material for: Qualitative and quantitative analysis data of the major constituents of Ilex paraguariensis leaves by UPLC-PDA and QTOF-MS
Source: Data Brief. 2016 May 20;8:295–9. doi: 10.1016/j.dib.2016.05.022 (PMC4906130; doi:10.1016/j.dib.2016.05.022)
Supplement: Supplementary file 1 — Supplementary material [file mmc1.docx]

Dear Editor

Prof. Sarah O'Loughlin

Herewith we are sending for publication our revised manuscript entitled **"Qualitative and quantitative analysis data of the major constituents of *Ilex paraguariensis* leaves by UPLC-PDA and QTOF-MS"** DIB-D-16-00299.

We agree with all of the observations raised by the Editor and the Reviewer (e-mail dated April 4^th^, 2016). The main changes in the manuscript are uploaded to editorial manager system.

We thank you for the efforts to improve the quality of the manuscript, explaining clearly the critical points to turn it proper for publication. We hope that the revised version is now suitable for publication in Data in Brief.

We kindly ask you to send acknowledgement of receipt and all correspondence to my address or to the e-mail.

All authors hereby declare that they have no conflict of interest to disclose. We hereby transfer, assign, or otherwise convey all copyright ownership, including any and all rights incidental thereto, exclusively to the journal, in the event that such work is published by the journal.

Best regards.

Flávio Henrique Reginatto, PhD.

Laboratório de Farmacognosia

Departamento de Ciências Farmacêuticas

Universidade Federal de Santa Catarina - UFSC

Campus Universitário/Trindade

Trindade - 88040-900 - Florianópolis, SC - Brazil

Telefone: +55 (48) 37215065
